# Cognitive Frailty as a Distinct Construct: Differential Risk Factors and Reversibility

**DOI:** 10.1002/alz.095266

**Published:** 2025-01-09

**Authors:** Laura Tay, Ee Ling Tay, Shimin Mah, YeeSien Ng

**Affiliations:** ^1^ Sengkang General Hospital, Singapore, Singapore Singapore; ^2^ Sengkang General Hospital, Singapore Singapore; ^3^ Singapore General Hospital, Singapore Singapore

## Abstract

**Background:**

Cognitive frailty (CF) has been proposed as a state of reduced cognitive reserve that is intermediate between age‐related cognitive changes and neurodegenerative diseases. We aim to examine (i) whether cognitive frailty presents with risk factors distinct from isolated phenotypes of physical frailty and cognitive impairment, (ii) reversibility of cognitive frailty subtypes.

**Methods:**

Participants were older adults aged >60 years from a community frailty screening programme. Physical frailty was assessed using Fried phenotype. Cognitive status was assessed based on response to a single question on memory symptoms and performance of the modified Chinese version of Mini‐Mental State Examination (CMMSE). All participants completed assessments for social vulnerability, mood, comorbidities, functional performance, sensory problems (hearing, vision), nutritional status, and sarcopenia. Each participant was classified into one of the diagnostic groups: reversible cognitive frailty (rCF) defined as subjective cognitive decline with prefrailty/ frailty; potentially reversible cognitive frailty (pCF) defined by mild cognitive impairment co‐existing with prefrailty/ frailty; (iii) isolated prefrailty/ frailty (PF); isolated cognitive impairment (CI); robust‐cognitively intact. Participants were followed at 1 year for frailty and cognitive status.

**Results:**

Among 961 participants, 351 (36.2%) were robust‐cognitively intact, 112 (11.7%) had rCF, 56 (5.8%) had pCF, 279 (29.0%) had isolated PF, and 163 (17.0%) had isolated CI. Depression increased risk for both CF subtypes (rCF: RRR 17.59; pCF: RRR 7.97; p<0.001), isolated PF [RRR 4.72 (2.76, 4.31)], and isolated CI [RRR 4.85 (2.11, 11.12)]. Sarcopenia was significantly associated with CF (rCF: RRR 15.12; pCF: RRR 14.20; both p<0.001) and isolated PF [RRR 16.13 (5.61, 46.40)]. Visual impairment was significantly associated with rCF [RRR 2.40 (1.26, 4.59)] and isolated CI [RRR 2.01 (1.16, 3.45)], while hearing impairment was associated with isolated PF [RRR 1.75 (1.00, 3.76)]. Education was protective against CF and isolated CI, while participation in social/ community activities was protective against PF only. At 1‐year, 25% of rCF and 12.5% of pCF reverted to being robust‐cognitively intact.

**Conclusion:**

Depression is a common risk factor across cognitive frailty subtypes and isolated phenotypes of physical frailty and cognitive impairment. Sarcopenia may contribute to cognitive frailty through physical frailty.

